# System-Wide Analysis Reveals a Complex Network of Tumor-Fibroblast Interactions Involved in Tumorigenicity

**DOI:** 10.1371/journal.pgen.1003789

**Published:** 2013-09-19

**Authors:** Megha Rajaram, Jinyu Li, Mikala Egeblad, R. Scott Powers

**Affiliations:** 1 Cancer Genome Center, Cold Spring Harbor Laboratory, Woodbury, New York, United States of America; 2 Graduate Program in Genetics, Stony Brook University, Stony Brook, New York, United States of America; 3 Cold Spring Harbor Laboratory, Cold Spring Harbor, New York, United States of America; University of Washington, United States of America

## Abstract

Many fibroblast-secreted proteins promote tumorigenicity, and several factors secreted by cancer cells have in turn been proposed to induce these proteins. It is not clear whether there are single dominant pathways underlying these interactions or whether they involve multiple pathways acting in parallel. Here, we identified 42 fibroblast-secreted factors induced by breast cancer cells using comparative genomic analysis. To determine what fraction was active in promoting tumorigenicity, we chose five representative fibroblast-secreted factors for *in vivo* analysis. We found that the majority (three out of five) played equally major roles in promoting tumorigenicity, and intriguingly, each one had distinct effects on the tumor microenvironment. Specifically, fibroblast-secreted amphiregulin promoted breast cancer cell survival, whereas the chemokine *CCL7* stimulated tumor cell proliferation while *CCL2* promoted innate immune cell infiltration and angiogenesis. The other two factors tested had minor (*CCL8*) or minimally (*STC1*) significant effects on the ability of fibroblasts to promote tumor growth. The importance of parallel interactions between fibroblasts and cancer cells was tested by simultaneously targeting fibroblast-secreted amphiregulin and the *CCL7* receptor on cancer cells, and this was significantly more efficacious than blocking either pathway alone. We further explored the concept of parallel interactions by testing the extent to which induction of critical fibroblast-secreted proteins could be achieved by single, previously identified, factors produced by breast cancer cells. We found that although single factors could induce a subset of genes, even combinations of factors failed to induce the full repertoire of functionally important fibroblast-secreted proteins. Together, these results delineate a complex network of tumor-fibroblast interactions that act in parallel to promote tumorigenicity and suggest that effective anti-stromal therapeutic strategies will need to be multi-targeted.

## Author Summary

There is increasing interest in developing methods to treat cancer by targeting non-cancer cells that play supportive roles in the tumor microenvironment. One type of non-cancer cell that has received considerable attention along these lines is cancer-associated fibroblasts, which can promote tumor formation and tumor growth. There have been several studies showing that inhibition of individual fibroblast genes or proteins dramatically reduces the tumor supportive function of fibroblasts. From the perspective of developing a therapeutic strategy, what remains unclear is whether the several different important factors discovered to date reflect the requirement of a multitude of fibroblast factors to promote tumorigenicity, or whether it reflects the diversity of the epithelial cancer cells and fibroblasts used in these different studies. Here, we addressed this question directly using a single system of fibroblasts and breast cancer epithelial cells. Importantly, we found that a multitude of fibroblast factors are indeed required to promote tumorigenicity, and that they have different effects on the tumor microenvironment. Furthermore, we found that inhibiting multiple fibroblast-secreted factors is more efficacious than blocking individual factors. These results suggest that fibroblasts and cancer cells act through multiple parallel pathways and that effective anti-stromal therapeutic strategies will need to be multi-targeted.

## Introduction

Solid tumors are aberrant tissues where stromal cell types co-develop with and influence cancer cells [1]. Significant epigenetic alterations and gene expression changes occur in stromal cells as tumors progress, and the stromal changes are as strikingly different as those observed in the cancer epithelial compartments [2]–[5]. Many of these stromal cell changes are elicited by factors secreted by cancer cells, such as vascular endothelial growth factor (*VEGF*; MIM: 192240), which helps recruit and induce proliferation of endothelial cells [6]. Cancer cells also secrete factors that alter surrounding fibroblasts, such as transforming growth factor (*TGF*)-*β* (MIM: 190180) which induces fibroblasts to differentiate into myofibroblasts and secrete collagen, thereby contributing to the abundant extracellular matrix often observed in epithelial tumors [7]. In addition to *TGF*-*β* factors secreted by cancer cells that influence stromal fibroblasts include platelet derived growth factor (*PDGF*; MIM: 173430), interleukin *(IL)-6* (MIM:147620), *IL1-*α (MIM: 147760), and *WNT1* inducible signaling pathway protein (*WISP*)-*1* and -2 (MIM: 603398 and 603399) [8], [9].

Tumor associated fibroblasts have been shown to promote cancer cell proliferation, angiogenesis, extracellular matrix (ECM) remodeling, inflammation, invasion and metastasis [10], [11]. Several fibroblast-secreted or membrane-bound factors that mediate these effects have been identified, including *CXCL12* (MIM: 600835), hepatocyte growth factor (*HGF*; MIM:142409), matrix metalloproteinase *MMP14* (MIM: 600754), osteopontin (MIM: 166490), *TGF-β*, and *CCL2* (MIM: 158105) [12]–[17].

Some basic underlying processes involved in regulating the interactions between the epithelial cancer cells and the stromal fibroblasts have been identified. For example, several fibroblast-secreted factors are inflammatory cytokines whose expression is driven by NF-kappaB-dependent transcription in a process similar to the senescent secretory phenotype observed in aging fibroblasts [18], [19]. Additionally, a study of how fibroblasts co-evolve with tumor cells determined that fibroblasts gradually implement two signaling loops, involving *TGF-β* and *CXCL12*, which act together through both autocrine and cross-signaling mechanisms [20]. What remains unclear is whether the different factors involved in cancer cell-fibroblast interactions reflects a requirement of a multitude of fibroblast factors acting in parallel to promote tumorigenicity, or whether it reflects the diversity of the approaches and systems used to identity important interactions. Here, we designed a study to explore how the entire repertoire of fibroblast-secreted factors that are induced by human cancer cells function as a whole and compared the factors. This systems-level study was designed to be complimentary to approaches that focus on single genes or single processes. Our study indicates that the majority of induced fibroblast-secreted factors play a role in promoting tumorigenicity and that they do so through diverse effects on the tumor tissue.

## Results

### Comparative genomic analysis of pro-tumorigenic fibroblast factors induced by breast cancer cells

We adapted previously used systems of human cancer cells and fibroblasts [21]–[23], by using fibroblast lines that were amenable for the use of stable RNA interference (shRNA) so the relevance of candidate mediators of tumor-stromal interactions could be tested. We determined using co-injection assays that two human fibroblast lines previously shown to promote tumorigenicity in other systems (HFFF2 and HFF1) were able to promote tumorigenicity of two basal breast cancer subtype cell lines, MDA-MB-231 and Cal51, whereas two other human fibroblast lines (Wi-38 and CCD1112Sk) were not (Figure S1). We used the two tumor-supportive fibroblast lines (HFFF2 and HFF1) as models for patient-derived breast carcinoma associated fibroblasts and the two non-supportive fibroblasts as models for normal breast tissue derived fibroblasts. Using fibroblasts cell lines allowed shRNA transfection, selection and subsequent validation of gene silencing, whereas patient-derived breast fibroblasts cannot be passaged in culture long enough for such manipulations (data not shown; Ahmet Acar, personal communication). To determine at which point the host murine fibroblasts replaced the co-injected human fibroblasts, we tagged the human fibroblasts with green fluorescent protein (GFP) so that we could visualize how long they survived ensconced within developing tumors. We found that after three weeks, the number of co-injected GFP-tagged human fibroblasts comprised roughly 20% of the tumor stroma as judged by co-localization of GFP and the fibroblast stromal marker alpha-smooth muscle actin (α-SMA) (Figure S2), but the relative contribution steadily declined from week 3 to week 8, although the GFP-fibroblasts never completely disappear. The level of α-SMA positive cells within tumor stroma persisted during this same time period (Figure S2).

**Figure 1:**
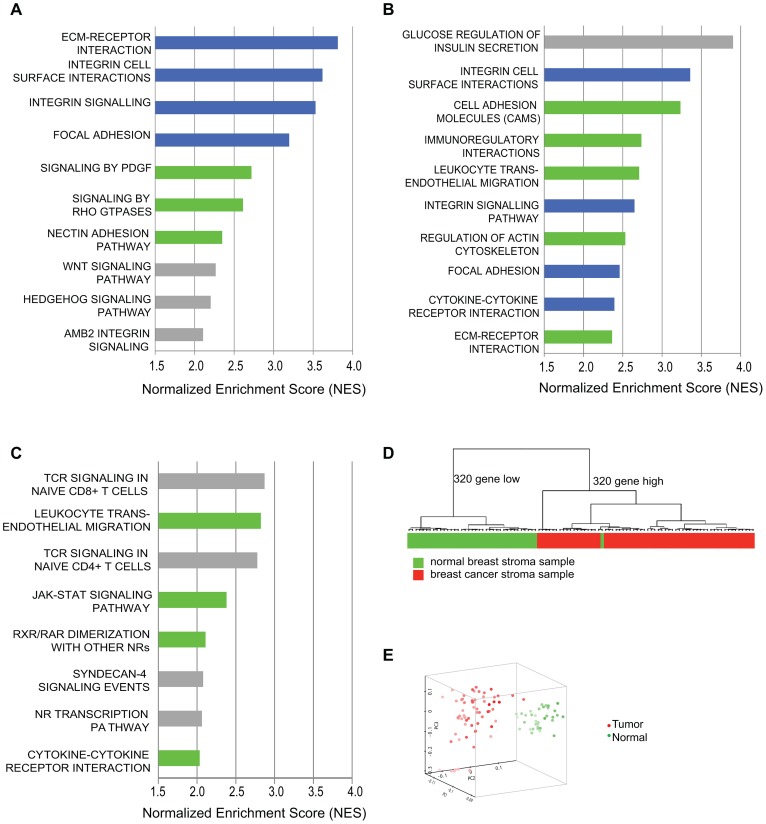
Comparative genomic analysis of expression changes induced by breast cancer cells in tumor-supportive fibroblasts, patient-derived carcinoma-associated fibroblasts, and microdissected breast stroma. (A) Top ten pathways identified by GSEA analysis of exposure of tumor-supportive fibroblasts to breast cancer cells; blue indicates overlap with the top ten pathways activated in patient-derived breast cancer fibroblasts; green indicates overlap with other pathways significantly activated in patient-derived breast cancer fibroblasts; grey indicates no overlap, (B) Top ten pathways identified by GSEA analysis of patient-derived breast cancer fibroblasts relative to their normal counterparts; blue indicates overlap with the top ten pathways activated by exposure of tumor-supportive fibroblasts to breast cancer cells; green indicates overlap with other pathways significantly activated by exposure of tumor-supportive fibroblasts to breast cancer cells; grey indicates no overlap, (C) Top ten pathways identified by GSEA analysis of microdissected breast cancer stroma relative to normal breast stroma, green indicates overlap with pathways significantly activated by exposure of tumor-supportive fibroblasts to breast cancer cells, but not in the top ten; grey indicates no overlap. (D) A tumor-supportive fibroblast gene signature was used in unsupervised clustering to classify normal (green) and tumor (red) microdissected breast stroma samples. (E) The same signature was used in principal component analysis to observe the separation of normal (green) and tumor (red) microdissected breast stroma samples.

We performed transcriptional profiling and pathway analysis to determine if exposure of our model tumor-supportive fibroblasts to breast cancer cells in co-culture resembled expression changes seen in patient-derived breast carcinoma fibroblasts. We compared expression profiles of HFF1 and HFFF2 tumor-supportive fibroblasts co-cultured with either Cal51 or MDA-MB-231 (four combinations total) to those of Wi-38 and CCD1112Sk non-supportive fibroblasts co-cultured with either Cal51 or MDA-MB-231 (another four combinations). We chose to work with basal subtype breast cancer cell lines due to the greater need to develop new treatments for this poor prognosis subtype. We then compared the pathways selectively enriched in tumor-supportive fibroblasts co-cultured with Cal51 and MDA-MB-231 to pathways enriched by comparing cultures of patient-derived breast carcinoma fibroblasts to their normal counterparts. Strikingly, the top four pathways identified by gene-set enrichment analysis (GSEA) that are activated by exposure of tumor-promoting human fibroblasts to breast cancer cells are also amongst the top ten pathways activated in cultures of patient-derived breast carcinoma fibroblasts relative to normal fibroblasts (Figure 1). These pathways are ECM-receptor interaction, focal adhesion, integrin signaling and integrin cell-surface interactions; interrelated pathways that have been shown to be involved in the activation of cancer associated fibroblasts [21], [24], [25]. Additionally, most of the other top ten activated pathways in both systems were activated in the other system at lower ranking but still significant levels. These pathways included cytokine cytokine-receptor interactions, *PDGF* signaling, and Rho GTPase signaling; which likewise have shown to be involved in activation of cancer associated fibroblasts [7], [13], [26] (Figure 1). On the whole, the overlap in all significantly activated pathways is 44% (Table S1). This affirmed that this system of tumor-promoting human fibroblasts resembled *in vitro* cultures of patient-derived breast cancer fibroblasts.

We next wanted to test whether this system also reflected changes observed *in vivo* in human primary breast cancer stroma. To address this, we used gene-set enrichment analysis to determine the pathways that were activated in microdissected human breast stroma relative to normal breast stroma [3]. Despite the fact that human breast stroma contains many cell types in addition to fibroblasts, four of the eight significantly activated pathways in human breast stroma were also significantly activated by exposure of tumor-promoting fibroblasts to breast cancer cells, including cytokine/cytokine-receptor interactions and JAK-STAT signaling (Figure 1). Additionally, we developed a gene signature based on stimulation of tumor-promoting fibroblasts by breast cancer cells. Based on both clustering and principal component analysis, this gene signature was able to correctly predict whether microdissected human breast stroma was derived from cancerous or normal breast tissue for 98 of 99 samples (Figure 1). These comparative genomic analyses establish the close correspondence of our system of interaction of breast cancer cells with tumor-promoting fibroblasts to stroma and stromal fibroblasts isolated from primary human breast cancer.

### Determining the repertoire of fibroblast-secreted factors that are induced by breast cancer cells

We used genome-wide analysis to determine the full repertoire of secreted factors induced by breast cancer cells in our tumor-promoting fibroblasts. As the first step, we compared genes induced in tumor-promoting fibroblast lines to genes induced in fibroblast lines incapable of promoting tumorigenicity. We found 320 genes that were more than 2-fold greater induced in tumor-promoting fibroblasts (Table S2) and within this group were 62 genes encoding secreted proteins. Of these, 42 were also significantly upregulated (p<0.05) in stromal cells isolated from primary breast cancer relative to normal breast stroma cells [3], [4], [27]. This group contained cytokines (15), extracellular matrix proteins (7), proteases (6), and growth factors and hormones (6) (Table 1). Cytokines as a class were significantly enriched in our screen relative to their representation in the set of all secreted proteins (36% vs. 12%, p = 5.6e-6). This enrichment is consistent with the prior reports that cytokines play key roles in the tumor-supportive function of tumor-associated fibroblasts [19]. In contrast, the other functional classes of secreted proteins were not significantly enriched. The cytokines upregulated in tumor-supportive fibroblasts included CC- and CXC-chemokines (*CCL-2, -5, -7, -8, -20*, and *CXCL-5, -10*; MIM: 158105, 187011, 158106, 602283, 600324 and 147310 respectively), pro-inflammatory interleukins (IL-1α, -1β, -8, -11, -24; MIM: 147760, 147720, 146930, 147681 and 604136 respectively), colony stimulating factor (*G-CSF*; MIM: 138970), interleukin receptor antagonist (*IL1RN*; MIM: 147679), and TNF superfamily member *TNSF15* (MIM: 604052) (Table 1). Systematic literature searches revealed that 16 of the 42 secreted proteins had one or more publication(s) implicating them in the tumor-supportive function of fibroblasts while 26 did not (Table 1). The genes not previously implicated as mediators of the tumor-supportive function of fibroblasts included the majority (8/15) of cytokines, one-half (3/6) of the proteases, and the majority (4/6) of the growth factors and hormones. Examples include *CCL8*, encoding a chemokine not previously linked to cancer that is involved in homing of memory T lymphocytes to inflamed skin [28]; pregnancy-associated plasma protein-a (*PPAPA*; MIM: 176385), a protease that degrades IGF-binding proteins and acts as a positive modulator of local IGF signaling in skin repair [29]; and *EGFL6* (MIM: 300239), encoding an epidermal growth factor (EGF) repeat protein expressed in osteoblastic-like cells and capable of inducing migration of endothelial cells [30].Some of the fibroblast-secreted candidate tumor-supportive factors fall outside of the known classes of proteins involved in tumor-stromal interactions, including *ISG15* (MIM: 147571), an interferon-inducible, ubiquitin-like protein whose secretion plays a critical role in mediating an effective immune response to mycobacteria [31]; and complement component *C3* (MIM: 120700), which in addition to its role in the complement cascade helps mobilize hematopoietic stem/progenitor cells to wounds [32].

**Table 1:** 42 secreted proteins selectively induced by breast cancer cells in tumor-promoting fibroblasts.

Gene Symbol	MIM ID	Description	Class	Cancer Relevant Phenotype	Known pro-tumorigenic stromal fibroblast function	Reference (PMID)
*CCL8*	602283	chemokine (C-C motif) ligand 8	Cytokine	Inflammatory	No	21217759
*TNFSF15*	604052	tumor necrosis factor superfamily, member 15	Cytokine	Suppressive	No	22833050
*CCL2*	158105	chemokine (C-C motif) ligand 2	Cytokine	Oncogenic	Yes	22472119
*CCL20*	601960	chemokine (C-C motif) ligand 20	Cytokine	Oncogenic	Yes	21847358
*CCL5*	187011	chemokine (C-C motif) ligand 5	Cytokine	Oncogenic	Yes	21252118
*CCL7*	158106	chemokine (C-C motif) ligand 7	Cytokine	Oncogenic	Yes	19937793
*CSF3*	138970	colony stimulating factor 3 (granulocyte)	Cytokine	Oncogenic	No	12492491
*CXCL10*	147310	chemokine (C-X-C motif) ligand 10	Cytokine	Oncogenic	No	17409450
*CXCL5*	600324	chemokine (C-X-C motif) ligand 5	Cytokine	Oncogenic	Yes	19047182
*FST*	136470	Follistatin	Other	Oncogenic	Yes	19740438
*IL11*	147681	interleukin 11	Cytokine	Oncogenic	No	12842083
*IL1A*	147760	interleukin 1, alpha	Cytokine	Oncogenic	No	9618523
*IL1B*	147720	interleukin 1, beta	Cytokine	Oncogenic	Yes	20138012
*IL1RN*	147679	interleukin 1 receptor antagonist	Cytokine	Suppressive	No	21519029
*IL24*	604136	interleukin 24	Cytokine	Suppressive	No	20926331
*IL8*	146930	interleukin 8	Cytokine	Oncogenic	Yes	22422937
*COL7A1*	120120	collagen, type VII, alpha 1	ECM	Suppressive	Yes	22564523
*SPON1*	604989	spondin 1, extracellular matrix protein	ECM	Oncogenic	No	19549008
*TNFAIP6*	600410	tumor necrosis factor, alpha-induced protein 6	ECM	Anti-inflammatory	No	9244409
*DPT*	125597	Dermatopontin	ECM	Oncogenic	No	16899568
*POSTN*	608777	periostin, osteoblast specific factor	ECM	Oncogenic	Yes	22158103
*VCAN*	118661	Versican	ECM	Oncogenic	Yes	19160015
*SPP1*	166490	secreted phosphoprotein 1	ECM	Oncogenic	No	21252118
*AREG*	104640	Amphiregulin	Growth factor	Oncogenic	No	18470483
*EGFL6*	300239	EGF-like-domain, multiple 6	Growth factor	None known	No	
*STC1*	601185	stanniocalcin 1	Growth factor	Oncogenic	No	20484106
*UCN2*	605902	urocortin 2	Growth factor	Suppressive	No	18308934
*NRG1*	142445	neuregulin 1	Growth factor	Oncogenic	Yes	22535374
*WISP1*	603398	WNT1 inducible signaling pathway protein 1	Growth factor	Suppressive	Yes	21516124
*C3*	120700	complement component 3	Other	HSPC recruitment	No	16863905
*FAM20C*	611061	family with sequence similarity 20, member C	Other	None known	No	
*FJX1*	612206	four jointed box 1 (Drosophila)	Other	None known	No	
*IGFBP5*	146734	insulin-like growth factor binding protein 5	Other	Suppressive	No	21460855
*ISG15*	147571	ISG15 ubiquitin-like modifier	Other	None known	No	
*PAPPA*	176385	pregnancy-associated plasma protein A, pappalysin 1	Protease	Oncogenic	No	21303951
*PCSK1*	162150	proprotein convertase subtilisin/kexin type 1	Protease	Oncogenic	No	20052009
*SERPINB2*	173390	serpin peptidase inhibitor, clade B (ovalbumin), member 2	Other	Suppressive	No	1427403
*SPINT1*	605123	serine peptidase inhibitor, Kunitz type 1	Other	Suppressive	No	16103220
*MMP1*	120353	matrix metallopeptidase 1 (interstitial collagenase)	Protease	Oncogenic	Yes	11990853
*MMP11*	185261	matrix metallopeptidase 11 (stromelysin 3)	Protease	Both oncogenic and suppressive	Yes	11990853
*MMP12*	601046	matrix metallopeptidase 12 (macrophage elastase)	Protease	Oncogenic	No	21378275
*MMP3*	185250	matrix metallopeptidase 3 (stromelysin 1, progelatinase)	Protease	Oncogenic	Yes	11990853

Results of literature searches on the 42 secreted proteins selectively induced in tumor-supportive fibroblasts. Descriptions are from Ingenuity (www.ingenuity.com). Semi-automated searches were performed using the gene symbols and different search terms (e.g. stromal fibroblast, tumor microenvironment, cancer) with PubMatrix (pubmatrix.grc.nia.nih.gov).

### Development of functional assays to test the effects of selected candidate mediators of fibroblasts on tumor growth and tumor microenvironment

**Figure 2:**
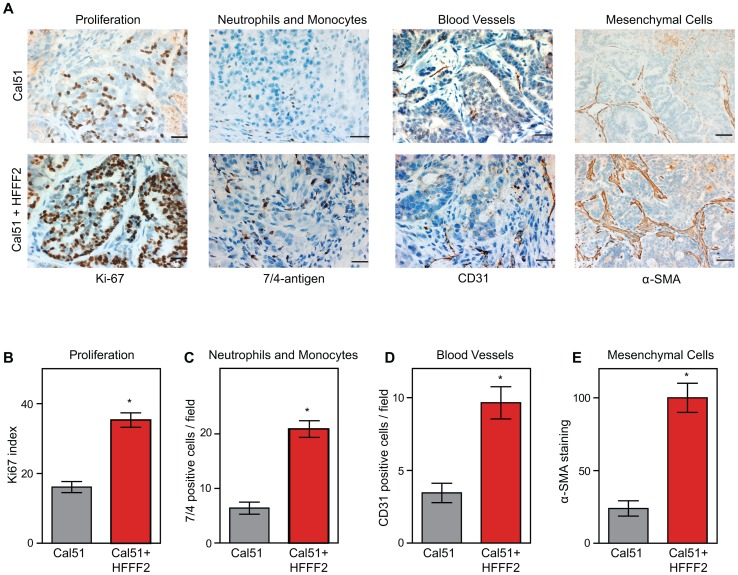
Tumor-supportive fibroblasts have profound effects on the composition of the tumor microenvironment. (A) Tumor-supportive fibroblasts HFFF2 increase Cal51 tumor cell proliferation and increase stromal components (bottom panel) as compared to tumors formed using Cal51 alone (top panel). Immunohistochemical analysis using antibodies to Ki-67 (proliferation), antigen #7/4 (neutrophils and monocytes), CD31 (blood vessels), and α-SMA (mesenchymal cells). Scale bars represent 50 µm for Ki-67, #7/4 and CD31 and 100 µm for α-SMA. (B) Quantification of Ki-67 positive (proliferating) tumor cells. Five different fields of three different tumors per group were scored. Asterisk indicates a significant difference between cell line only (Cal51shNT) and co-injection (Cal51shNT+HFFF2) groups (p<0.01). Data are expressed as the mean ± SEM. (C–E) Similarly performed quantification of monocytes and neutrophils, endothelial cells, and activated mesenchymal cells (p<0.01).

In order to characterize the effects of fibroblasts on tumor progression, we co-injected tumor-promoting fibroblasts and examined their effects on both tumor cells and associated non-tumor cells within the tumor microenvironment. Consistent with a faster tumor growth rate, cancer cells in the co-injected tumors exhibited a two-fold higher proliferative rate based on Ki-67 labeling (Figure 2). To visualize and quantify the presence of three of the most important features of the tumor microenvironment - inflammation, vascularization and fibroblast activation - we performed immunohistochemical analysis at week six after co-injection. We used antibodies to the 7/4-antigen, which is highly expressed in neutrophils and inflammatory monocytes [33], the CD31 endothelial antigen, which visualizes blood vessels [34], and α-SMA, which is expressed by activated fibroblasts [35]. Strikingly, the presence of inflammatory cells, degree of vascularization, and number of activated fibroblasts were all 3 to 4 fold higher in the co-injected tumors (Figure 2), indicating a profound influence of tumor-supportive fibroblasts on the composition of the tumor microenvironment.

We decided to analyze a mix of previously implicated and novel candidate pro-tumorigenic fibroblast-secreted factors. Additionally, we wanted to test whether seemingly redundant factors were indeed functionally redundant. We focused on cytokines, growth factors, and hormones, and chose *CCL2* and *CCL7* (both previously validated as functionally important fibroblast secreted factors [17], [36]); the related factor *CCL8* which, like *CCL7*, binds *CCR1* (MIM: 601159); amphiregulin (*AREG*; MIM: 104640), which has been implicated in tumor stromal-interactions but as a ligand produced by cancer cells acting on fibroblasts [37]; and stanniocalcin1 (*STC1*; MIM: 601185), which has been shown to act as a cancer cell autonomous factor, but not as a stromally produced factor [38]. For each of these five genes, we confirmed by quantitative RT-PCR that there was a significant induction in the co-cultured tumor supportive fibroblasts relative to the co-cultured neutral fibroblasts (Figure S3).

### *CCL2*, *CCL7* and *CCL8* chemokines mediate tumor supportive abilities of fibroblasts with distinct effects on the tumor microenvironment

**Figure 3:**
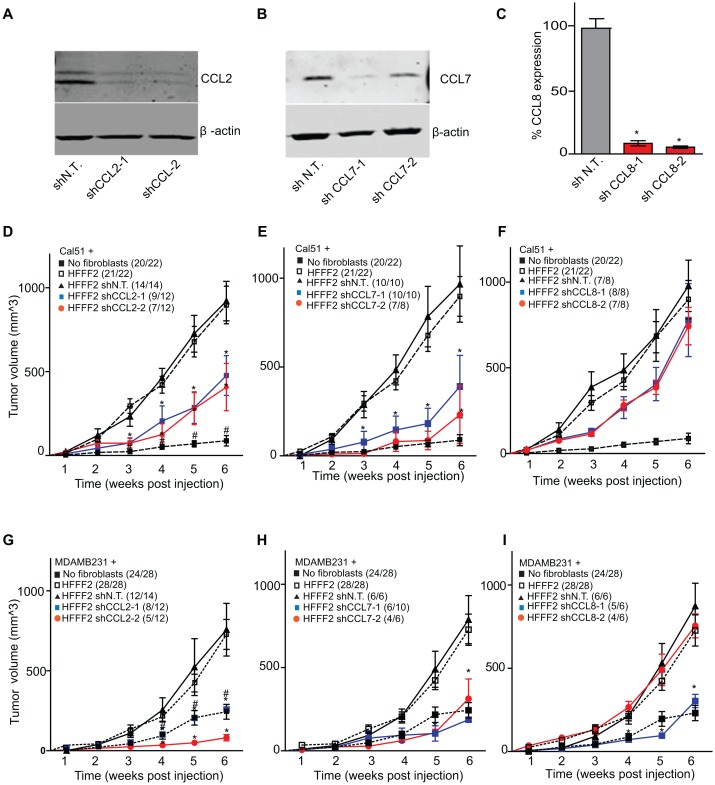
shRNA silencing of *CCL2*, *CCL7* or *CCL8* in tumor-supportive fibroblasts reduces their ability to promote tumorigenicity. (A) Reduction of CCL2 protein in HFFF2 fibroblasts by expressing shRNAs targeting *CCL2* as indicated compared to non-targeting (N.T.) control determined by immunoblotting. Beta-actin was used as a loading control. (B) Reduction of CCL7 protein levels in HFFF2 fibroblasts expressing shRNAs targeting *CCL7*. (C) Reduction in *CCL8* RNA levels in HFFF2 fibroblasts expressing shRNAs targeting *CCL8* compared to HFFF2 fibroblasts expressing non-targeting (N.T.) control. Asterisk indicates a significant difference (p<0.01) between the experimental (shCCL8) and control (shN.T.) group. (D) Tumorigenicity of Cal51 cells alone or co-injected with HFFF2 cells expressing either no shRNA, control shRNA or shRNAs targeting *CCL2*. Tumor-take rate for the total injections for each group is indicated. Asterisks indicate significant differences (p<0.01) in tumor volumes for shCCL2-1 and shCCL2-2 compared to control. Hashtag indicates a significant difference (p<0.05) between cell line only (Cal51) and Cal51 co-injected with HFFF2 fibroblasts. Error bars represent SEM. (E) Effects of shRNAs targeting *CCL7* (p<0.01). There was no significant difference in the tumor volumes between the two knockdown groups (p = 0.3, 0.3, 0.2 and 0.5 at weeks 3, 4, 5 and 6 post injections respectively). (F) Effects of shRNAs targeting *CCL8* (p<0.01). (G) Tumorigenicity of MDAMB231 cells alone or co-injected with HFFF2 cells expressing either no shRNA, control shRNA or shRNAs targeting *CCL2*. Tumor-take rate for the total injections for each group is indicated. Asterisks indicate significant differences in tumor volumes for shCCL2-1 and shCCL2-2 compared to control (p<0.01). Hashtag indicates a significant difference between cell line only (MDAMB231) and MDAMB231 coinjected with HFFF2 fibroblasts (p<0.05 and n = 10 per group). Errors represent SEM. (H) Effects of shRNAs targeting *CCL7* (p<0.01). (I) Effects of shRNAs targeting *CCL8* (p<0.01). The lack of effects of the CCL-targeting shRNAs on fibroblast proliferation are shown in Figure S4.

**Figure 4:**
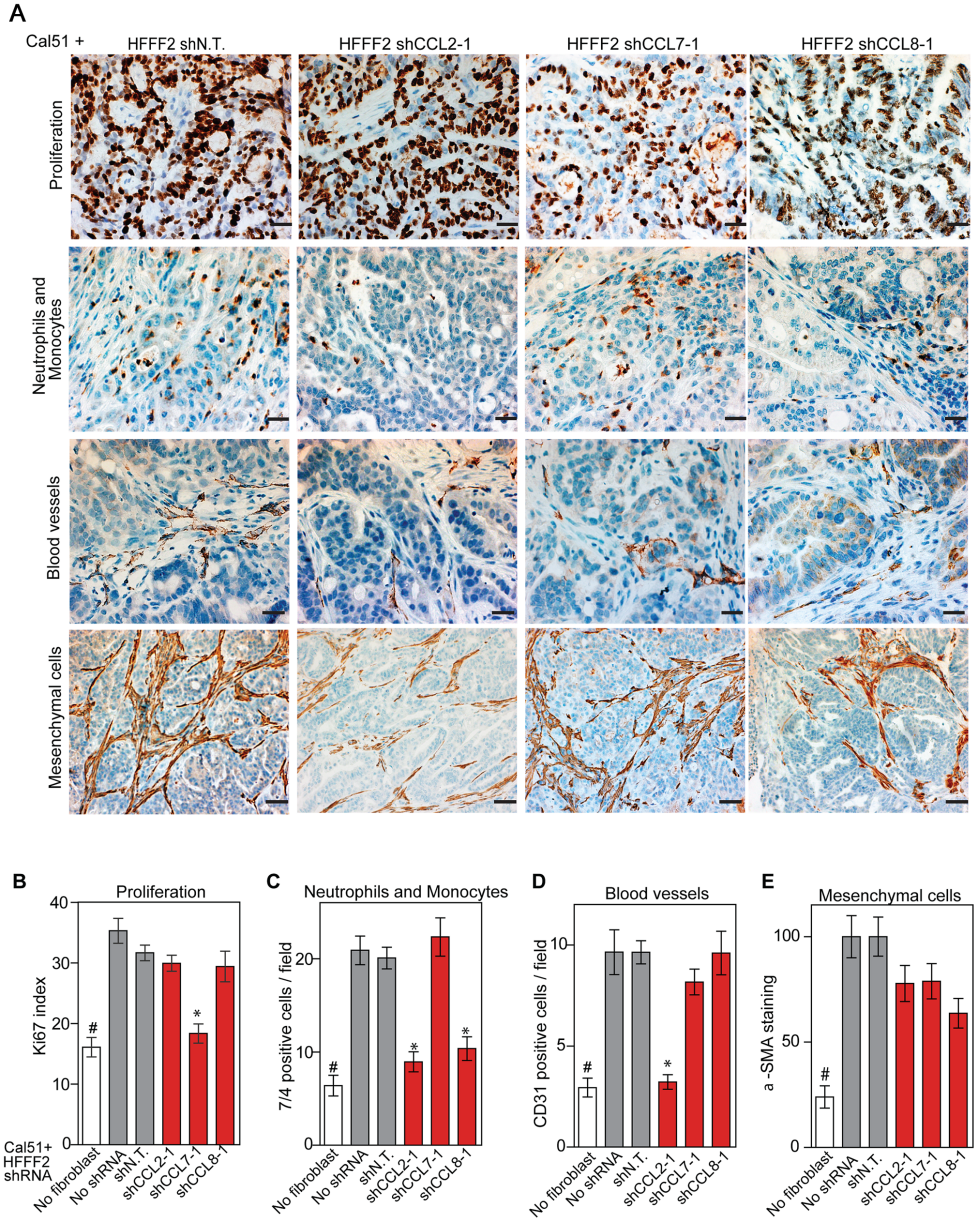
Diverse effects on the tumor microenvironment mediated by fibroblast secretion of the related chemokines CCL2, CCL7 and CCL8. (A) Immunohistochemical analysis of the effects of suppressing *CCL2*, *CCL7* or *CCL8* in tumor-supportive fibroblasts on cancer cell proliferation, immune cell recruitment, blood vessel recruitment and mesenchymal cell activation in Cal51 tumors, using antibodies to Ki-67 (proliferation), antigen #7/4 (immune cells; neutrophils and monocytes), CD31 (endothelial cells of blood vessels) and α-SMA (mesenchymal cell activation). Scale bars represent 50 µm for Ki-67, antigen #7/4 and CD31 panels and 100 µm for α-SMA. (B–E) Quantification of tumor cell proliferation, monocytes and neutrophils, blood vessel endothelial cells, and mesenchymal cell activation. For each property, five different fields of three different tumors per group were scored. Data are expressed as the mean ± SEM. Asterisks indicates a significant difference between the experimental shRNAs and control non-targeting shRNA (p<0.01). # indicates a significant difference in proliferation in Cal51 only group compared to Cal51 coinjected with HFFF2 fibroblasts (p<0.01). Data are expressed as the mean ± SEM.

CCL2, CCL7, and CCL8 are structurally related chemokines that share a common function of recruitment of monocytes to areas of injury and inflammation [39]. Based on their structural similarity and overlapping functions in inflammation, we wanted to determine if they had redundant roles in the tumor supportive function of co-injected fibroblasts. Remarkably, shRNAs directed against each of these three cytokines suppressed tumorigenicity in the co-injection assay, with the strongest effects observed when silencing *CCL2* (53%) or *CCL7* (66%) compared to weaker effects exerted by silencing of *CCL8* (25%) (two validated shRNAs per gene, Figure 3). Interestingly, despite their related structure, silencing of each of the three cytokines had a distinct impact on the tumors: silencing of *CCL2* suppressed recruitment of innate immune cells and angiogenesis (Figure 4) almost to the same levels as in tumors without co-injected fibroblasts. In contrast, silencing of *CCL8* suppressed only the recruitment of innate immune cells, while silencing of *CCL7* reduced tumor cell proliferation almost to the levels observed in tumors growing in the absence of co-injected fibroblasts (Figure 4 and Figure S4). Our data therefore showed that these related chemokines had non-redundant roles in mediating fibroblast-supportive functions.

### Suppressing *AREG* reduces the ability of fibroblasts to support tumorigenicity

**Figure 5:**
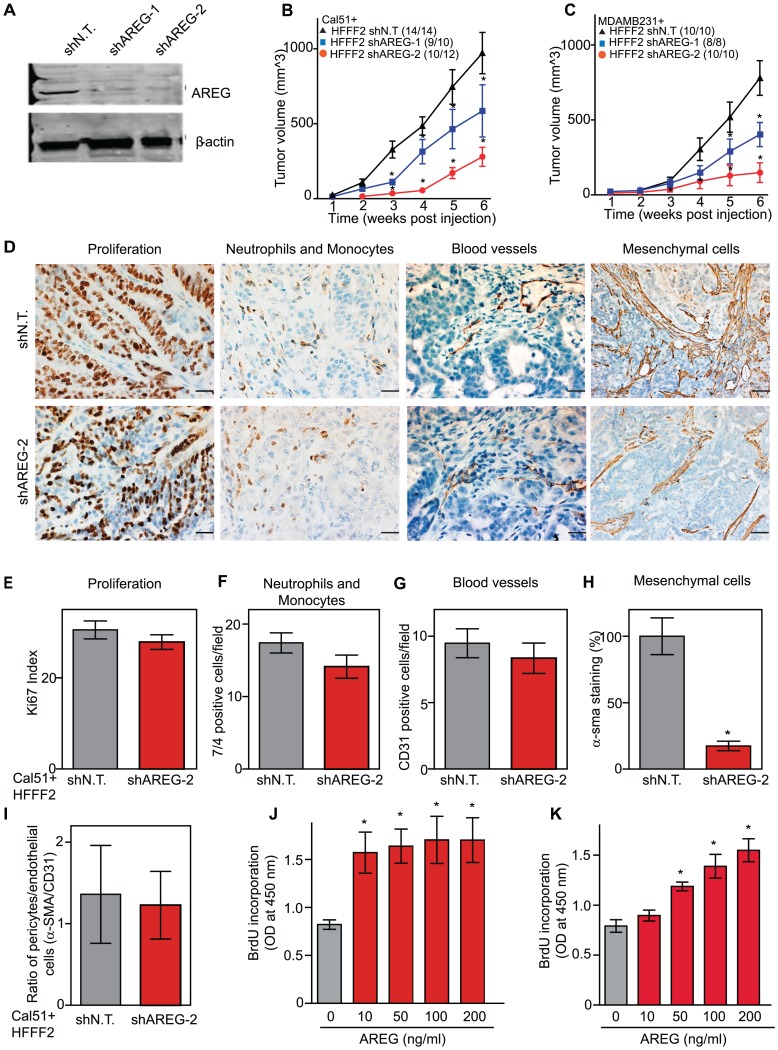
Suppressing amphiregulin expression in tumor-supportive fibroblasts reduces tumorigenicity and amount of mesenchymal cells. (A) Reduced amphiregulin protein levels in HFFF2 fibroblasts expressing two distinct shRNAs directed against *AREG* compared to cells expressing non-targeting shRNA (immunoblotting). β-actin was used as a loading control. shAREG-2 was more potent at suppression both by immunoblotting and quantitative RT-PCR (see also Figure S3). (B) Reduced tumorigenicity of Cal51 cells co-injected with HFFF2 cells expressing either shRNAs targeting *AREG* as compared to control shRNA. Tumor-take rates for each group are indicated. Asterisks denote significant differences in tumor volumes for shAREG-1 or shAREG-2 compared to control (p<0.05) Data are expressed as the mean ± SEM. (C) As with (B) but for MDA-MB-231 breast cancer cells. p<0.05. (D) Immunohistochemical analysis of the effects of suppressing amphiregulin expression in tumor-supportive fibroblasts HFFF2 on Cal51 tumor cell proliferation and the tumor microenvironment (bottom panel) compared to tumors formed using Cal51 co-injected with control HFFF2 (top panel) as described in the legend to Figure 2. (E) Quantification of Ki-67 positive tumor cells in Cal51 tumors injected with control HFFF2 fibroblasts or shAREG-2 HFFF2 fibroblasts. Five different fields of three different tumors per group were scored. Error bars represent SEM. There was a small decrease in proliferation in the shAREG-2 HFFF2 fibroblast group (not statistical significance, p = 0.06). (F) Similarly performed quantification of monocytes and neutrophils, with a slight decrease observed in the shAREG-2 HFFF2 fibroblast group (not, significant, p = 0.14). (G) Similarly performed quantification of blood vessel endothelial cells, with no significant difference (p = 0.24). (H) Similarly performed quantification of activated mesenchymal cells, asterisk indicates a significant difference (p = 0.002). (I) Pericyte coverage of tumor associated blood vessels in tumors derived from injection of Cal51 cells with HFFF2 fibroblasts expressing shRNA-targeting *AREG* compared to control. Fifteen vessels from three tumors were examined for each group were analyzed for the ratio of pericytes (α-SMA^+^) to endothelial cells (CD31^+^). Data are expressed as the mean ± SEM. (J) The effect of amphiregulin on the replicative rate of wild-type mouse embryonic fibroblasts (WT-MEFs) measured by quantifying BrdU incorporation. Data representative of three independent experiments are shown. Concentration of amphiregulin (AREG ng/ml) is indicated on the x-axis. Asterisk indicates significant differences (p<0.05) between the control (0 ng/ml AREG) and experimental groups (10, 50, 100 and 200 ng/ml AREG). (K) As in J, but assaying the replicative rate of human fibroblasts, HFFF2.

**Figure 6:**
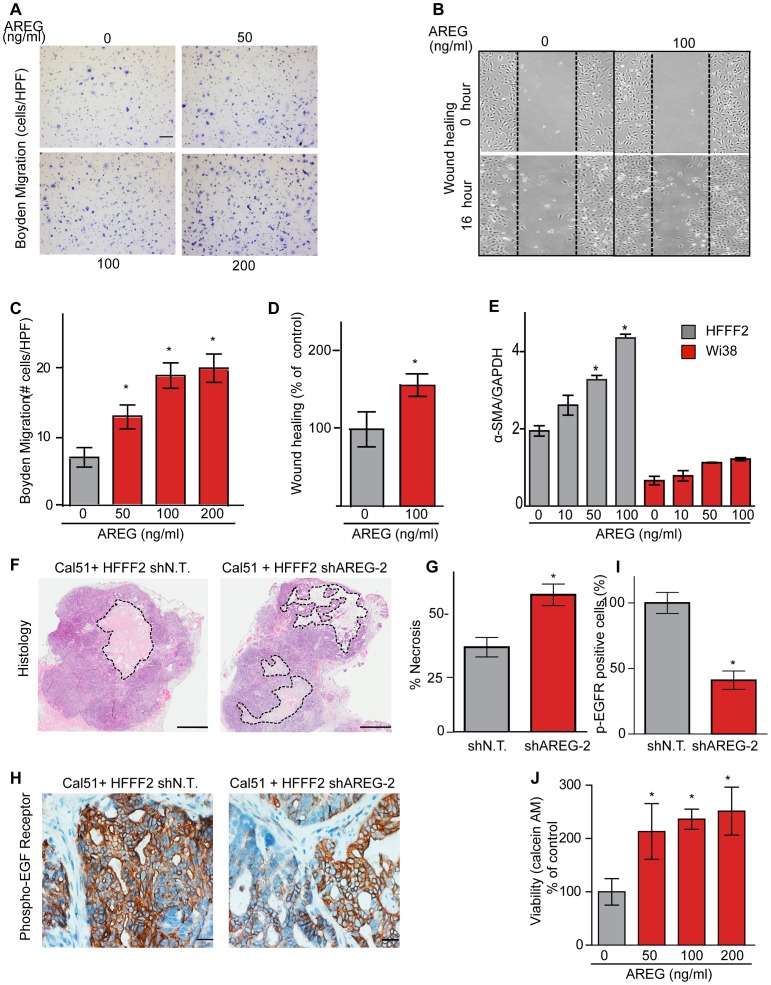
Amphiregulin is a chemoattractant for fibroblasts and helps prevent necrosis and tumor cell death. (A) Photomicrographs of mouse fibroblasts that have transversed a Boyden chamber in response to different concentrations of amphiregulin in the opposing chamber. Migration was measured 5 hours post plating. Scale bars represent 100 µm. (B) Amphiregulin promotes migration of mouse embryonic fibroblasts (MEFs) in a scratch wound-healing assay (photomicrographs at indicated time after initiation of assay). (C) Quantification of the Boyden migration assay. Data are expressed as the mean ± SEM. Asterisks indicate a significant difference (p<0.05) between experimental and control groups. (D) Quantification of migration in scratch wound healing assay (cells that had moved into the scratched area as percent of control). Asterisk indicates a significant difference between the control and experimental group (p<0.05). (E) Quantitative RT-PCR analysis of α-SMA expression (relative to GAPDH) in HFFF2 fibroblasts (gray bars) and Wi38 fibroblasts (red bars) upon treatment with amphiregulin. Asterisk indicates a significant difference between untreated HFFF2 fibroblasts and those treated at 50 and 100 ng/ml (p value 0.01 and 0.001, respectively). All data are expressed as the mean ± SEM. (F) Necrosis in Cal51+HFFF2 tumors with control shRNA or with shRNA targeting *AREG* as visualized by H&E staining. Dashed lines indicate necrotic areas. Scale bars represent 1 mm. (G) Quantification of necrosis in Cal51+HFFF2 tumors with control shRNA or with shRNA targeting *AREG*. Necrotic area was calculated from five different tumors per group. Data are expressed as the mean ± SEM. Asterisks indicate a significant difference (p = 3e-4) between experimental and control groups. (H) EGFR activation (phosphorylation) in Cal51+HFFF2 tumors with control shRNA or with shRNA targeting *AREG* detected by immunostaining using an antibody to phospho-EGFR (Tyr1068). Scale bars represent 50 µm. (I) Quantification of phospho-EGFR positive tumor cells in Cal51+HFFF2 tumors with control shRNA or with shRNA targeting *AREG*. Areas positive for pEGFR were calculated from five different fields of five different tumors per group. Asterisk indicates that the experimental group is significantly different than the control (p = 0.007). Data are expressed as the mean ± SEM. (J) Effects of amphiregulin on the viability of Cal51 cells plated on non-adhesive plates and cultured for 24 hours (anoikis assay). Viability was determined by measuring calcein AM uptake. Asterisk indicates that the experimental group is significantly different than the control (p<0.05). Data are expressed as the percentage of viable cells normalized to control with the error bars representing SEM.

We next tested whether silencing of *AREG* in fibroblasts affected tumor supportive function. Silencing of *AREG* had a pronounced effect, with an average reduction in tumor size at six weeks of 55% to 65% (Figure 5). In contrast to the chemokines, silencing *AREG* in fibroblasts had no effect on the number of blood vessels or innate immune cells (Figure 5). However, immunohistochemical analysis of the percentage of cells within the tumor that expressed α-SMA, a marker of mesenchymal-derived cells such as activated fibroblasts, revealed a 78% reduction. This level was comparable to the low levels of α-SMA positive cells in tumors formed in the absence of co-injected fibroblasts (Figure 5). Although α-SMA is expressed by activated pericytes, a cell type that surrounds endothelial cells of blood vessels, we did not observe a difference in number of α-SMA cells associated with blood vessels (Figure 5). This suggested that secretion of amphiregulin by the co-injected human fibroblasts plays a major role in establishing a tumor microenvironment that is enriched for activated fibroblasts, and since the co-injected human fibroblasts are almost entirely replaced by mouse fibroblasts at the point of excision (6 weeks), this effect must be propagated through recruited mouse fibroblasts. Consistent with this, we found that amphiregulin increased proliferation of mouse fibroblasts, as well as human fibroblasts (Figure 5). We also found that amphiregulin was a chemoattractant for mouse fibroblasts in migration and invasion assays, suggesting a mechanism of recruitment into the tumor, and that amphiregulin directly activated fibroblasts as judged by induction of α-SMA expression (Figure 6).

In addition to the pronounced reduction of activated fibroblasts when *AREG* was silenced, we also noted changes in activation of the amphiregulin receptor EGFR (MIM: 131550) on tumor cells. Compared to tumor cells co-injected with control tumor-supportive fibroblasts, tumor cells when co-injected with *AREG*-silenced fibroblasts showed a <2-fold reduction of activated, phospho-EGFR as measured by immunohistochemistry (Figures 6). This was accompanied by a minor, barely significant (p = 0.06), negative effect on tumor cell proliferative rate *in vivo*, with no direct effect of amphiregulin on tumor cell proliferation *in vitro* (Figure 5 and Figure S5). In contrast, we found a significant increase in necrosis in tumors after silencing of fibroblast-secreted amphiregulin (Figure 6). Notably, amphiregulin significantly protected the breast cancer cells from cell death induced by detachment from their normal extracellular matrix (anoikis, Figure 6). Together, these results suggest that fibroblast-secreted amphiregulin has potent effects on tumor progression, with autocrine effects leading to activation of fibroblasts and paracrine effects protecting cancer cells from cell death.

### Strong tumor suppressive responses are achieved by simultaneously targeting different mediators of tumor-fibroblast interactions on both cell types

**Figure 7:**
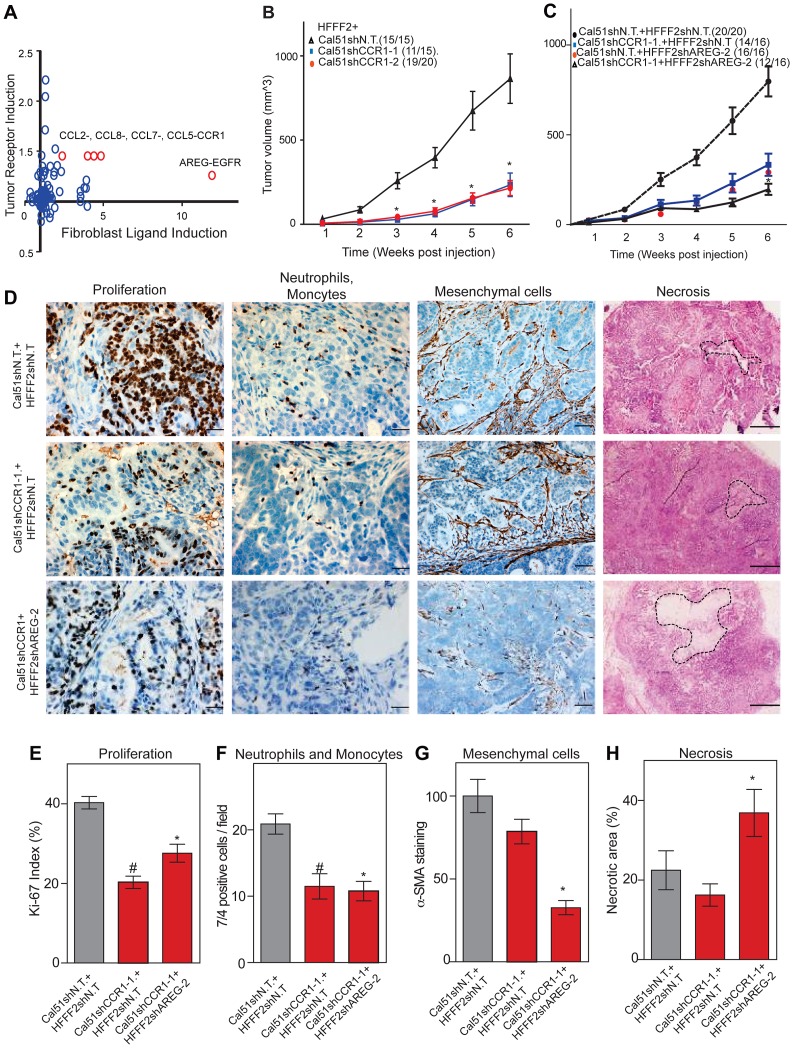
Combined inhibition of chemokine and amphiregulin signaling is more effective at blocking the effects of tumor supportive fibroblasts. (A) Paracrine upregulation of the expression of ligand-receptor pairs upon co-culture of tumor supportive fibroblasts with basal breast cancer cells. The fold-change in ligand expression in tumor-supportive fibroblasts (x-axis) is plotted along with the fold-change in receptor expression in breast cancer cells (y-axis). (B) Tumorigenicity of Cal51 cells expressing either control shRNA or shRNAs targeting *CCR1* co-injected with HFFF2 fibroblasts. Tumor take rate for each group is indicated. Asterisks indicate significant differences in tumor volumes for shCCR1-1 and shCCR1-2 co-injection groups compared to control (p<0.01) (C) Tumorigenicity of Cal51 and HFFF2 fibroblasts coinjected in the following combinations: Cal51 cells expressing shRNAs targeting *CCR1* co-injected with HFFF2 fibroblasts expressing control shRNA; Cal51 cells expressing control shRNA coinjected with HFFF2 cells expressing shRNA targeting AREG; Cal51 cells expressing shRNAs targeting *CCR1* co-injected with HFFF2 fibroblasts expressing shRNA targeting AREG. Tumor take rate for each group is indicated. Asterisks indicate significant differences (p<0.05). (D) Histological and Immunohistochemical analysis of the effects of suppressing CCR1 in Cal51 cells and amphiregulin in tumor-supportive fibroblasts HFFF2 as described in the legend to Figure 2 but also tumor necrosis which was evaluated on hematoxylin and eosin stained sections. Scale bars represent 50 µm for Ki67 and 7/4, 100 µm for α-SMA and 500 µm for hematoxylin and eosin staining. (E) Quantification of microenvironment effects as described in the legend to Figure 2. Five different fields of three different tumors per group were scored. Error bars represent SEM. Hashtag indicates a significant difference in proliferation in the CCR1 silenced group compared to the control where p = 8.9E-04. Asterisk indicates a significant difference in proliferation in the CCR1 and AREG silenced group compared to the control where p = 0.01. (F) Similarly performed quantification of neutrophils and monocytes where Hashtag indicates a significant difference in the CCR1 silenced group compared to the control where p = 4.6E-04. Asterisk indicates a significant difference in the CCR1 and AREG silenced group compared to the control where p = 2.4E-05. (G) Similarly performed quantification of activated mesenchymal cells. Asterisk indicates a significant difference in the CCR1 and AREG silenced group compared to the control where p = 0.003. (H) Similarly performed quantification of necrotic area. Asterisk indicates a significant difference in the CCR1 and AREG silenced group compared to the control where p = 0.02.

Co-culturing also lead to changes in gene expression in the breast cancer cells, which upregulated a shared receptor for *CCL2* and *CCL7*, the chemokine receptor *CCR1* upon co-culturing with tumor-supportive fibroblasts (Figure 7). We therefore tested whether *CCR1* expression by cancer cells was critical for some of the tumor supportive functions of fibroblasts by stably expressing shRNAs targeting *CCR1* in Cal51 breast cancer cell line (knockdown efficiency was quantified by both qRT-PCR and immunoblotting, Figure S6). Silencing of *CCR1* had no effect on tumor growth when cancer cells were injected alone. However, in the context of co-injection with fibroblasts, silencing of cancer cell *CCR1* resulted in a 3-fold reduction in tumor size, almost eliminating the effect of the fibroblasts (Figure 7 and Figure S6). We further observed a 40% reduction in the proliferative index of cancer cells and a 2-fold reduction in recruitment of neutrophils and inflammatory monocytes to the tumor, but only minor effects on the number of blood vessels and mesenchymal cells (Figure 7 and Figure S6). Thus, expression of *CCR1* by cancer cells plays a critical role in enabling fibroblasts to exert tumor supportive function, through increased tumor cell proliferation and potentially indirectly through recruitment of leukocytes.

We next asked whether blocking two interactions between fibroblasts and cancer cells was more effective than blocking either one alone. We therefore asked whether simultaneously silencing fibroblast-secreted amphiregulin and cancer cell expressed *CCR1* was more efficacious than blocking either alone. We found that tumor growth was significantly more reduced when both pathways were targeted (Figure 7).

### Cancer cell induction of the fibroblast-secreted proteins requires parallel activation

A number of studies have implicated specific cancer-cell secreted factors in the activation of neighboring fibroblasts, including *TGF-β* [40] and *IL-1β* [19]. We wanted to determine whether the repertoire of tumor-promoting fibroblast secreted factors could be induced by single specific inducers, or whether multiple pathways were acting in parallel. To test this, we used quantitative RT-PCR to measure gene expression changes in tumor-promoting fibroblasts of the five factors (*CCL-2, -7, -8, AREG*, and *STC1*) that we had determined all had functional relevance, along with two others factors (*NRG1*; MIM: 142445 and *WISP1*) that also were induced in the system. Surprisingly, *TGF-β*, did not induce expression of any of the seven factors (Table 2), despite its ability to induce activation of fibroblasts to myofibroblasts, which resembles many aspects of carcinoma-associated fibroblasts [10]. *AREG* also did not induce expression of any of the seven factors, despite its key role in stimulating mammary fibroblasts during normal mammary development [37]. *IL-1β*, a potent activator of *NF-*κ*B* signaling, produced an upregulation in chemokines *CCL-2, -7, -8* similar to that seen by co-culture with breast cancer cells (Table 2). However, *IL-1β* did not induce expression of *WISP1, STC1, AREG, or NRG1*. Interestingly, a combination of *IL-1β* and *AREG* lead to significant upregulation of *WISP1* in addition to stimulation of *CCL-2, -7, -8*. In contrast, none of the fibroblast factors were induced when *TGF-β* and *IL-1β* were combined (Table 2). Based on these results, it seems likely that the ability of breast cancer cells to induce the full spectrum of pro-tumorigenic fibroblast-secreted proteins involves a multitude of interacting factors, including some not previously identified. We also found that co-culture of tumor-promoting fibroblasts with a normal, non-malignant breast epithelial cell line, MCF10A, was able to induce fibroblast expression of two out of the seven factors induced by breast cancer cells, namely *AREG* and *WISP2* (Table 2). For these two factors, it would appear that breast epithelial cells *per se*, regardless of tumorigenic properties, elicit the same response in fibroblasts. This result is consistent with the key role that stromal *AREG* plays in normal mammary development [37].

**Table 2:** Induction in fibroblasts of chemokines and growth factors after treatment with single or combination of factors.

Treatment	Fold induction						
	CCL2	CCL7	CCL8	WISP1	STC1	AREG	NRG1
AREG	1.0	1.3	0.7	1.6	0.9	1.0	1.1
IL1*β*	6.3*	6.3*	60.4*	1.0	1.7	1.1	1.3
TGF*β*	0.7	1.9	1.9	1.4	0.3	0.6	1.9
IL1*β*+AREG	10.1*	7.8*	55.3*	4.0*	2.0	1.4	1.4
IL1*β*+TGF*β*	1.3	2.5	0.9	1.9	0.4	0.6	2.0
TGF*β*+AREG	0.4	1.6	ND	1.9	0.2	0.7	1.9
AREG+IL1*β*+TGF*β*	1.0	2.0	0.3	1.3	0.4	0.9	2.1*
MCF10A co-culture	1	1.1	1.1	2.1*	0.9	6.1*	0.9
Cal51 co-culture	5.7*	4.8*	63.7*	2.2*	7.0*	9.7*	2.3*
MDAMB231 co-culture	6.2*	4.0*	114.8*	3.6*	11.8*	23.5*	4.0*

Induction of chemokines (CCL2, CCL7 and CCL8) and growth factors (AREG, NRG1,WISP1 and STC1) in HFFF2 fibroblasts co-cultured with breast cancer cells or normal breast epithelial cells (co-culture Cal51; co-culture MDAMB231; MCF10A co-culture) or upon treatment with individual factors IL1*β* (10 ng/ml), AREG (100 ng/ml) or TGF*β* (10 ng/ml) or a combination was measured by qRT-PCR. Fold induction was calculated as the change in gene expression compared to untreated HFFF2 control cells. GAPDH was used as the normalization control for all experiments. Data shown is average of three experiments. Asterisk indicated that the fold induction is significant; p<0.05.

## Discussion

Numerous studies have used the co-injection assay to identify the factors produced by fibroblasts that are responsible for promoting tumorigenicity. These studies have identified single factors that when inhibited strongly suppress the ability of activated fibroblasts to promote tumorigenicity, and they include scatter factor, *SDF-1*, *MMP14*, *NF-*κ*B*, *osteopontin*, *TGF-β*, and *CCL2* [12]–[17]. However, these studies did not address whether the single factors were unique in their capacity of promoting fibroblast-supported tumorigenicity, nor did they compare different factors. Here, we used comparative genomics to identify 42 candidate mediators of fibroblast-promoted tumorigenicity and we tested the functional impact of a set of five of these factors. Surprisingly, we found that four of the five tested factors promoted tumorigenicity, three of them strongly (*CCL7*, *CCL2*, and *AREG*) and one of them weakly (*CCL8*) (Table 3). Although the fifth factor *STC1* significantly affected tumorigenicity by the area under the tumor growth curve test (Table 3), it failed to show significant effects using t-tests at any single time points (data not shown). Since we only tested five of the 42 fibroblast-secreted-factors that were induced by breast cancer cells, it seems highly likely that an even greater number of fibroblast-secreted factors play a role in promoting tumorigenicity. Thus, our study indicates that even in a single system there are a large number of secreted factors involved in the ability of fibroblasts to promote carcinomas, rather than a single important mediator.

**Table 3:** Comparison of the effects on tumorigenicity and the microenvironment of inhibiting different fibroblast-secreted factors.

Cal51+shHFFF2	Control	shAREG	shCCL2	shCCL8	shCCL7	shSTC1
**Area under curve**						
Mean	1	0.37	0.27	0.58	0.26	0.60
SEM	0.01	0.03	0.02	0.02	0.03	0.03
pval		<0.01	<0.01	<0.01	<0.01	0.01
**Endpoint tumor volume**						
Mean	1	0.41	0.43	0.71	0.34	0.89
SEM	0.01	0.02	0.03	0.03	0.03	0.04
p value		<0.01	<0.01	0.04	<0.01	0.46
**Tumor microenvironment**						
Proliferation	1	0.91	0.95	0.93	0.58	NA
p value		0.06	0.35	0.43	<0.01	
Immune cells	1	0.81	0.33	0.52	1.11	NA
p value		0.14	<0.01	<0.01	0.34	
Blood vessels	1	0.88	0.44	1	0.85	NA
p value		0.24	<0.01	0.98	0.11	
Mesenchymal cells	1	0.17	0.77	0.63	0.78	NA
p value		<0.01	0.08	0.051	0.11	

Summary of results obtained from tumorigenicity assays and immunohistochemical characterization of the tumor microenvironment in AREG, CCL2, CCL7, CCL8 and STC1 silenced tumors compared to non-target control. Three major parameters, namely total area under the curve (AUC), endpoint tumor volume (week 6 post injection) and immunohistochemistry of tumor microenvironment were used to quantify differences between the control and experimental groups.

Intriguingly, our results also indicate widely diverse mechanisms for fibroblast-secreted factors in the promotion of tumorigenicity. The strong effects of fibroblast *CCL7* appeared to be caused by a significant effect on cancer cell proliferation (Table 3), which was unique among the factors tested. We also found that reducing cancer cell expression of the *CCL7* receptor, *CCR1*, also reduced fibroblast-induced proliferation. In contrast, the strong tumor promoting effects of fibroblast *CCL2* was associated with different effects on the tumor microenvironment, as we found significant decreases in both angiogenesis and recruitment of innate immune cell upon silencing of *CCL2*. *CCL8* had weaker effects on tumor growth than the other tested chemokines, which likely reflects it inability to affect either tumor proliferation or vascularity (Table 3). Fibroblast *AREG* also had very strong effects on tumor growth, and influenced both the total number of activated fibroblasts in the tumors and the survival of the cancer cells, a combination not observed with any other tested factor. Interestingly, secretion of amphiregulin by fibroblasts appeared to potentially act as a chemoattractant to recruit new fibroblasts and induce their proliferation and activation, resulting in a tumor microenvironment that is enriched in activated fibroblasts. Thus, in a single system of carcinoma cells and tumor-supportive fibroblasts, several factors play key roles.

Our study also sheds light on how cancer cells modify the stromal cells to enable them to promote tumorigenicity. We tested several cancer cell-secreted factors, previously reported to influence stromal cells, but none of them were able to induce the full panel of verified tumor-promoting fibroblast factors as well as the cancer cells themselves. Even in one simple system, it appears that cancer cells act on fibroblasts through multiple factors, resulting in the secretion of another complex set of factors that influence cancer cells and other components of the tumor microenvironment.

One of the key findings of our study was that inhibiting multiple interactions between cancer cells and fibroblasts is more efficacious than blocking individual pathways. This finding is not completely unexpected in light of the complexity of the tumor microenvironment and in fact previous reports have suggested this as a possibility [20], [41]. Nevertheless, this result highlight that the different interactions are non-redundant and act in parallel, but it also suggests that effective anti-stromal fibroblast therapeutic strategies can be achieved by taking a multi-targeted approach. Future research towards this end will need to employ models that closely resemble the type of tumors to be targeted in human patients. This presents many challenges, including the ability to selectively target endogenous fibroblasts in tumor tissues, along with the ability to monitor the in vivo response of tumor-associated fibroblasts. Despite these challenges, our study shows that there are several potential combinatorial targets for future fibroblast-targeted therapeutic approaches.

## Materials and Methods

### Data files

All genomic data for this study, including expression analysis of both fibroblasts and breast cancer cells, have been deposited in the Gene Expression Omnibus (GEO) repository (GSE41678). http://www.ncbi.nlm.nih.gov/geo/query/acc.cgi?token=bnepxkssyoamgdu&acc=GSE41678

### Cell lines, co-culture, flow cytometry and gene expression analysis

Breast cancer cell lines and human fibroblast strains were obtained from ATCC (Manassas, VA), DSMZ (Braunschweig, Germany) or Sigma (St.Louis, MO) and grown under standard tissue culture conditions in growth medium recommended by the supplier. For dual-color co-culture experiments, breast cancer cells stably expressing *Discosoma sp.* red fluorescent protein (DsRed) and human fibroblasts stably expressing *Zoanthus sp.* green fluorescent protein (ZsGreen) were generated using pRetroX-IRES-DsRedExpress vector and pRetroX-IRES-ZsGreen1 vector (Clontech, CA) respectively via retroviral transduction (detailed description in Supplemental Experimental Procedures). None of the experiments utilized multiple retroviral transfections. For the co-culture experiments, we transfected the original fibroblasts with a fluorescent marker in order to facilitate cell separation before transcriptome analysis (the breast cancer cells were transfected with a different fluorescent marker). For the shRNA experiments, we transfected the original fibroblasts with validated shRNA constructs from the Broad library. Co-culture of fluorescently-tagged breast cancer cells and fibroblasts was initiated by plating 1.5 million fibroblasts into 10 cm dishes, and after 18 hours 1 million breast cancer cells were added and then incubated for six days. Monocultures were performed in parallel for the same duration. Following co-culture or mono-culture, cells were trypsinized and resuspended in FACS sorting buffer (PBS+1% FBS) for separation into DsRed+ and ZsGreen+ populations using an ARIA II flow cytometer and analyzed using FACS DiVA software (Becton Dickenson, CA). Total RNA was isolated using RNeasy kit (Qiagen, Netherlands) and hybridized to Gene 1.0 ST arrays (Affymetrix, CA). Data was extracted, background corrected, normalized, and converted from probe values to gene values using the AROMA R package (www.aroma-project.org).

### Tumorigenicity assays

All studies utilizing human xenograft experiments were approved by and in accordance with Cold Spring Harbor Laboratory's Institutional Animal Care and Use Committee. Five to six week old female nude mice (NCR nu/nu; Charles River Inc., Wilmington, MA) were irradiated at 400 cGy 24–36 hours prior to injections. One million breast cancer cells were trypsinized, resuspended with or without 1.5 million fibroblasts in 100 µl DMEM and injected subcutaneously into both flanks of irradiated, nude mice. Growth was followed over time by taking caliper measurements at indicated time points. Tumor volume was measured as 0.52×length×width^2^. Tumors were excised six-eight weeks post injections or when one of the measurements reached 2 cm.

### Immunostaining and quantification

Immunostaining procedure is described in detail in Supplementary Experimental Procedures. The primary antibodies used for immunostaining are as follows: α-SMA (1∶2000; # 1A4; Sigma-Aldrich), CD31 (1∶100; ab28364; Abcam), antigen 7/4 (1∶400, CL8993AP, Cedarlane), Ki-67 (1∶2000; MIB5; Dako), pEGFR (1∶100; 1138-1; Epitomics) and GFP (1∶1000; ab290; Abcam). Immunostained slides were quantified by counting (for 7/4, Ki-67 and CD31), by percentage of stained area (for α –SMA and pEGFR) using Image J software (NIH, Bethesda, MD).

Additional methods are found in the file Text S1.

## Supporting Information

Figure S1 Identification of tumor-supportive fibroblasts for basal breast cancer cells. (A–D) The effect of four different human fibroblast lines on the ability of the basal breast cancer cell line Cal51 to form tumors in nude mice. From left to right, the tumorigenic effects of co-injection of fibroblasts HFFF2 or HFF1, followed by the effects of co-injecting fibroblasts CCD1112Sk and Wi-38. Asterisks mark groups that are significantly different, p<0.05, n>10 per group. Data are expressed as the mean ± SEM. (E–H) Identifiably performed experiments with another basal breast cancer cell line, MDA-MB-231.
(EPS)

Figure S2 Time course study of the presence of co-injected HFFF2 fibroblasts in tumors formed with Cal51 cells. (A) The presence of GFP tagged human HFFF2 fibroblasts at different times after co-injection with Cal51 cancer cells. Activated fibroblasts were visualized with a red fluorescently labeled antibody to α-SMA and tissue counterstained with DAPI. Scale bars represent 100 µm. (B) the presence of HFFF2 fibroblasts is more easily visualized without labeling for α-SMA.
(TIF)

Figure S3 Quantitative RT-PCR validation of selective induction in tumor-supportive fibroblasts of candidate stromal mediators upon co-culture of basal carcinoma cells. (A) Quantitative RT-PCR validation of the selective up regulation *AREG* upon co-culture with tumor-supportive fibroblasts (HFF1 and HFFF2) versus tumor-neutral fibroblasts (Wi-38 and CD1112SK). “+” indicates co-culture with the indicated breast cancer cell line. Data are expressed as the mean ± SEM. (B) As in (A) but measuring *CCL2*. (C) As in (A) but measuring *CCL7*. (D) As in (A) but measuring *CCL8*. (E) As in (A) but measuring *STC1*.
(EPS)

Figure S4 Effects of shRNA silencing of *CCL2*, *CCL7* or *CCL8* on the tumor-supportive function of co-injected fibroblasts. (A) Quantitative RT-PCR validation of shRNA suppression of *CCL7* in HFFF2 fibroblasts. Asterisk indicates significant differences between expression of *CCL7* between the control (shN.T.) and the knockdown groups (p<0.05). Error bars represent SEM. No significant difference was observed in the expression of CCL7 between the two knockdown groups (p = 0.21). (B) The effects of shRNA suppression of *CCL2* on the viability of HFFF2 fibroblasts were determined using an MTT assay 48 hours post plating. No significant effects were observed. n = 6; Error bars represent SEM. (C) As in (B) but testing the effects of *CCL7* suppression. (D) As in (B) but testing the effects of *CCL8*.
(EPS)

Figure S5 Validation of shRNAs targeting *AREG* and effects of amphiregulin on tumor-cell proliferation. (A) *AREG* expression in HFFF2 fibroblasts expressing either control or shRNAs targeting *AREG*. GAPDH expression was used as a reference. Asterisk indicates a significant difference in *AREG* expression between control and shAREG-1, 2 and 3 (p = 0.016, 0.014 and 0.02 respectively). Data are expressed as the mean ± SEM. (B) Relative viability of HFFF2 fibroblasts expressing control or shRNAs targeting *AREG* as assayed by MTT following 48 hours of culture. Data are expressed as the mean ± SEM. (C) Proliferation of breast cancer cells (Cal51; gray bars or MDA-MB-231; red bars) was assayed by MTT following 72 hours of culture with the indicated amounts of amphiregulin. Data are expressed as mean ± SEM.
(EPS)

Figure S6 Combined shRNA suppression of *CCR1* and *AREG* blocks tumor-supportive function of co-injected fibroblasts. (A) Quantitative RT-PCR validation of shRNA suppression of *CCR1* in Cal51 breast cancer cells as well as demonstration that shRNAs targeting *CCR1* suppress protein levels in Cal51 cells. (B) The effects of shRNA suppression of *CCR1* on the viability of Cal51 cells was determined using an MTT assay 48 hours post plating. (C) Tumorigenicity of Cal51 cells expressing either control shRNA or shRNAs targeting *CCR1*. Tumor take rate for each group is indicated. No significant differences were observed between the groups. Errors represent SEM. (D) Immunohistochemical analysis of the tumor microenvironmental effects of suppressing tumor cell *CCR1* on blood vessel recruitment. Cal51 cells expressing control shRNA or shRNA targeting CCR1 were coinjected with HFFF2 fibroblasts expressing control shRNA or Cal51 expressing shRNA to CCR1 were injected with HFFF2 fibroblasts expressing shRNA to AREG. Scale bars represent 50 µm. (E) Quantification of blood vessel recruitment in tumor groups presented in (D). No significant difference was observed between the groups.
(TIF)

Table S1 Significantly activated pathways in the three tumor-stromal datasets.
(XLS)

Table S2 320 genes that were more than 2-fold greater induced in tumor-promoting fibroblasts.
(XLSX)

Text S1 Supplemental methods.
(DOCX)
