# Supplementary figures and images for: System-Wide Analysis Reveals a Complex Network of Tumor-Fibroblast Interactions Involved in Tumorigenicity
Source: PLoS Genet. 2013 Sep 19;9(9):e1003789. doi: 10.1371/journal.pgen.1003789 (PMC3778011; doi:10.1371/journal.pgen.1003789)

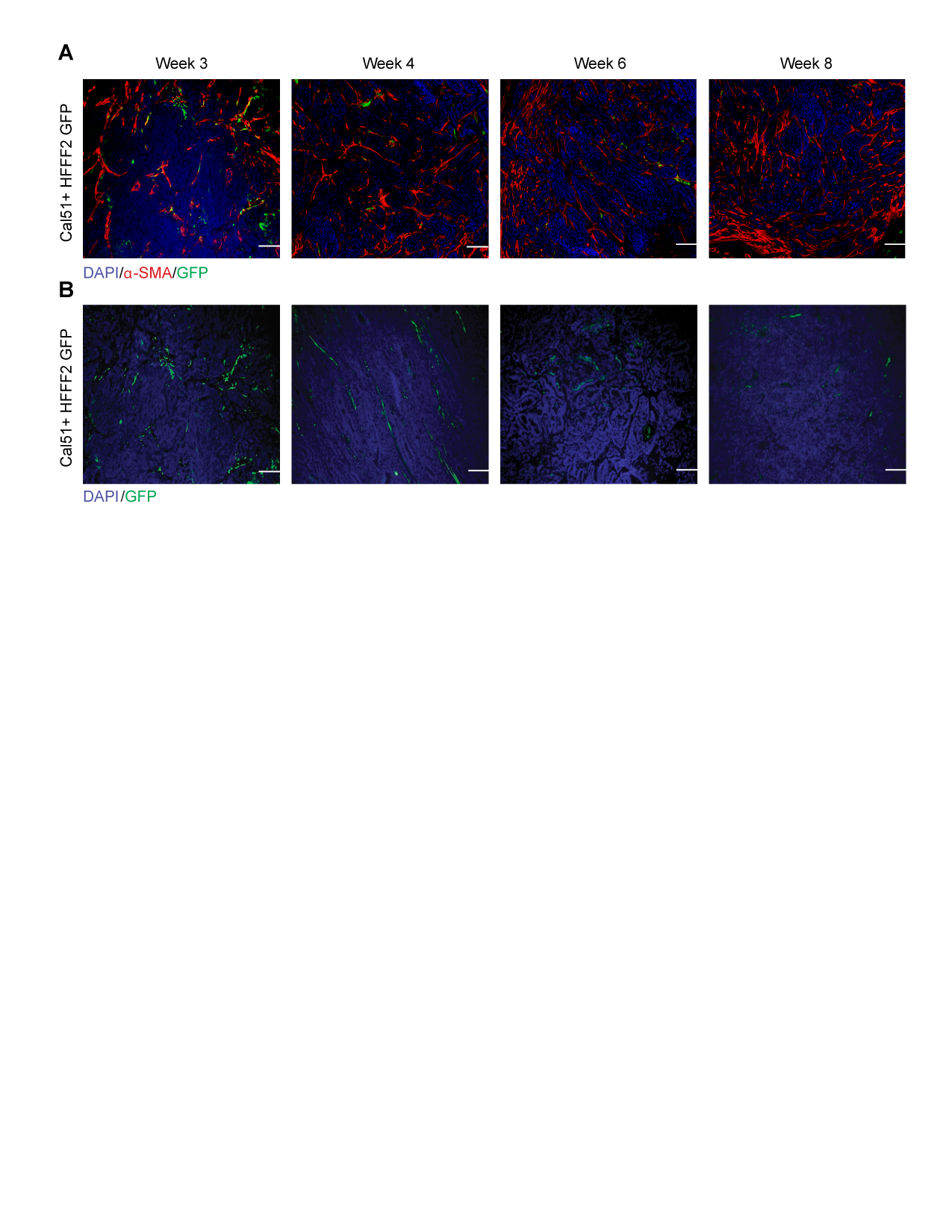

Supplement: Figure S2 — Time course study of the presence of co-injected HFFF2 fibroblasts in tumors formed with Cal51 cells. (A) The presence of GFP tagged human HFFF2 fibroblasts at different times after co-injection with Cal51 cancer cells. Activated fibroblasts were visualized with a red fluorescently labeled antibody to α-SMA and tissue counterstained with DAPI. Scale bars represent 100 µm. (B) the presence of HFFF2 fibroblasts is more easily visualized without labeling for α-SMA. (TIF) [file pgen.1003789.s002.tif]

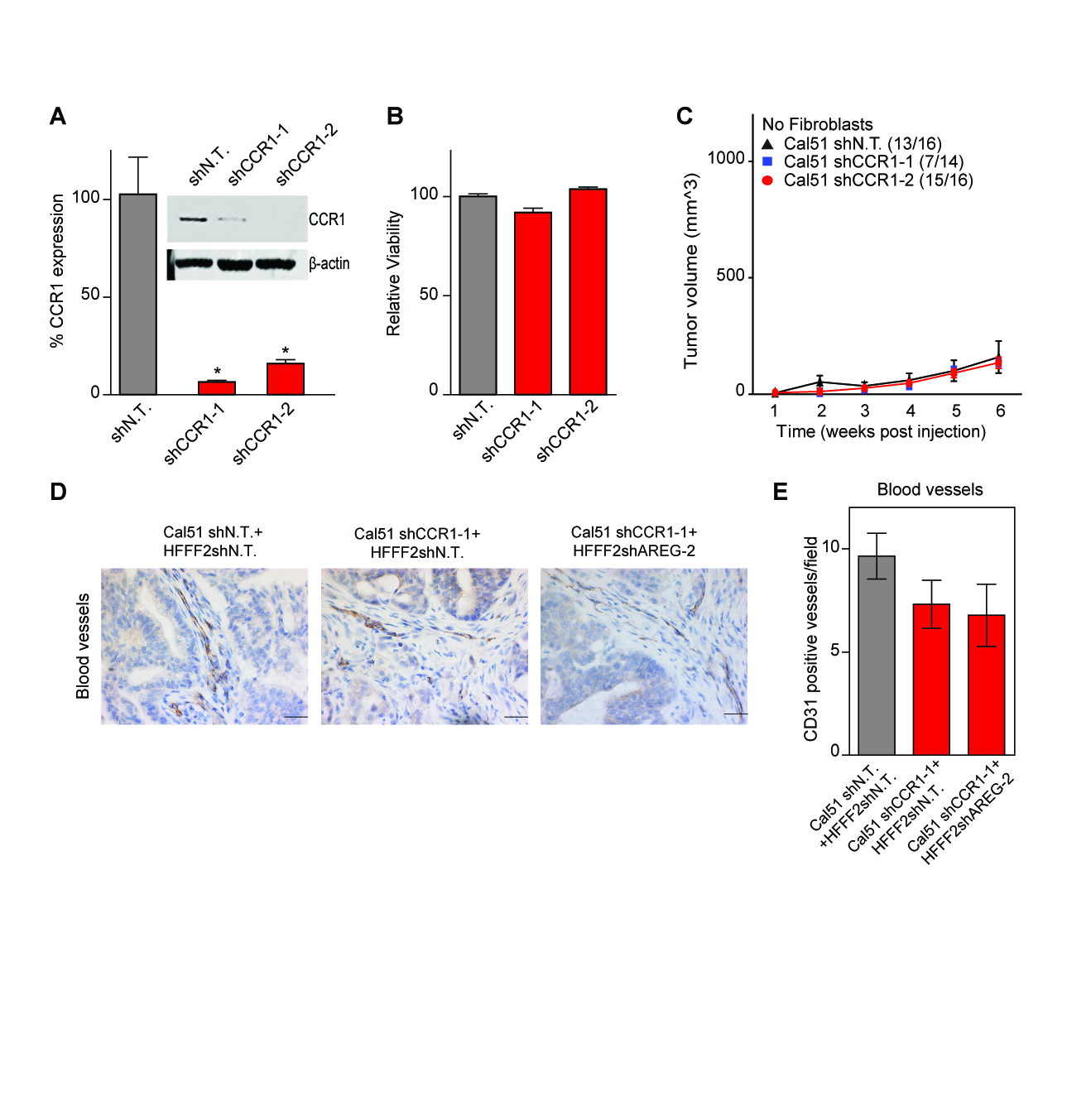

Supplement: Figure S6 — Combined shRNA suppression of CCR1 and AREG blocks tumor-supportive function of co-injected fibroblasts. (A) Quantitative RT-PCR validation of shRNA suppression of CCR1 in Cal51 breast cancer cells as well as demonstration that shRNAs targeting CCR1 suppress protein levels in Cal51 cells. (B) The effects of shRNA suppression of CCR1 on the viability of Cal51 cells was determined using an MTT assay 48 hours post plating. (C) Tumorigenicity of Cal51 cells expressing either control shRNA or shRNAs targeting CCR1. Tumor take rate for each group is indicated. No significant differences were observed between the groups. Errors represent SEM. (D) Immunohistochemical analysis of the tumor microenvironmental effects of suppressing tumor cell CCR1 on blood vessel recruitment. Cal51 cells expressing control shRNA or shRNA targeting CCR1 were coinjected with HFFF2 fibroblasts expressing control shRNA or Cal51 expressing shRNA to CCR1 were injected with HFFF2 fibroblasts expressing shRNA to AREG. Scale bars represent 50 µm. (E) Quantification of blood vessel recruitment in tumor groups presented in (D). No significant difference was observed between the groups. (TIF) [file pgen.1003789.s006.tif]
