# Supplementary material for: System-Wide Analysis Reveals a Complex Network of Tumor-Fibroblast Interactions Involved in Tumorigenicity
Source: PLoS Genet. 2013 Sep 19;9(9):e1003789. doi: 10.1371/journal.pgen.1003789 (PMC3778011; doi:10.1371/journal.pgen.1003789)
Supplement: Text S1 — Supplemental methods. (DOCX) [file pgen.1003789.s009.docx]

**Supplementary Information**

**Supplementary Tables**

Supplementary Table 1 (Excel File attached)

Supplementary Table 2 (Excel File attached)

**Supplementary Figures**

**Figure S1** (file attached)

**Figure S2** (file attached)

**Figure S3** (file attached)

**Figure S4 (file attached)**

**Figure S5 (file attached)**

**Figure S6 (file attached)**

**Figure S7 (file attached)**

**Supplemental methods**

**Membrane separated co-cultures**

Co-cultures of breast cancer cells and fibroblasts utilized 0.4 μm membrane inserts (Becton Dickenson, CA) fitted into 6-well tissue culture plates. 50,000 Cal51 or MDA-MB-231 were plated in the upper chambers while 150,000 fibroblasts (HFFF2, HFF1, Wi38 or CCD1112Sk) were plated in lower chambers and allowed to incubate for six days. Following this, the cells were washed, trypsinized and collected for RNA isolation.

**Computational methods**

Selection of the 320 genes preferentially induced in tumor-supportive fibroblasts during coculture was based on genes that were induced two-fold or more in tumor-supportive fibroblasts than neutral fibroblasts upon co-culture with Cal51 and MDA-MB-231. Heatmaps showing induction of the 320 genes were generated with heatmap.2 function in the gplots R package. To determine whether the 320 genes could predict whether human breast stroma was derived from tumor or normal tissues, we derived a gene signature using the principal component analysis of the labdsv R package. The first three component vectors were used to cluster tumor and normal samples from microdissected human breast stroma datasets and to separate the NKI dataset into samples with high overall expression of the 320 genes and samples with low expression. To determine paracrine induction of ligand-receptor pairs in co-cultured cancer epithelial cells and fibroblasts, we first determined which upregulated ligand receptor pairs that showed a significant (p < 0.05) positive correlation between ligand expression in fibroblasts and receptor expression in cancer epithelial cells, and then used GraphPad to plot the fold difference in induction between tumor-supportive fibroblasts and neutral fibroblasts.

**Histological analyses**

Excised tumors were rinsed in saline, stored in 10% PBS buffered formalin, processed through graded ethanol, paraffin embedded and cut into 10 μm thick sections. For histology analyses, formalin-fixed, paraffin-embedded sections were deparaffinized, rehydrated, and stained with Mayer’s hematoxylin and eosin. Necrosis analysis was performed using hematoxylin and eosin stained sections and necrotic area was calculated as a percentage of total area for each tumor section. For immunohistochemical analyses, paraffin-embedded tissue sections were deparaffinized, rehydrated and processed for antigen retrieval by heating sections in 1% citrate buffer in a pressure cooker for 20 minutes. Prior to incubation with the primary antibody, endogenous peroxidase activity was quenched using 3% hydrogen peroxide (Sigma, MO) except for sections being processed for immunofluorescence. Tissue sections were treated to block non-specific binding using 10% normal goat serum (Vector Labs, CA) for 30 minutes at room temperature. For immunostaining involving α−SMA, tissues were blocked with Mouse-on-Mouse blocking kit (Vector Labs). Following this, sections were incubated with the primary antibody overnight at 4^0^C. All immunostaining experiments were visualized using horseradish peroxidase-conjugated secondary antibodies with 3,3'-Diaminobenzidine (DAB) substrate (Vector Labs) and counterstained with Mayer’s hematoxylin.

**Retroviral infections, shRNA targeting and lentiviral transduction**

For retroviral infections, 1 million BING amphotropic packaging cells (ATCC) were transfected with 10 μg of retroviral plasmid (pRetroX-IRES-DsRedExpress/ZsGreen1; Clontech) and 5 μg of helper plasmid in 10-cm dishes using the Profection® Mammalian Transfection System (Promega, WI). After 48 hours, viral supernatant was collected, filtered using a 0.4 micron filter, supplemented with 5 μg/mL polybrene (Millipore, CA) and added to the target cells. Successful infection was determined by ~90% cells expressing fluorescent protein 48 hour post infection. shRNAs targeting candidate genes and non-targeting control shRNA (shN.T.) were obtained from The RNAi Consortium of the Broad Institute library (Sigma Aldrich) as bacterial stocks except shRNAs targeting CCR1 which were obtained as readymade lentiviral particles. shRNAs used to target candidate genes are as follows: shAREG1-4 (TRCN0000117994, TRCN0000117995, TRCN0000117993 and TRCN0000117996 respectively); shCCL2-1 and 2 (TRCN0000338418 and TRCN0000006283 respectively); shCCL7-1 and 2 (TRCN0000057893 and TRCN0000057894 respectively); shCCL8-1 and 2 (TRCN0000057948 and TRCN0000057949 respectively); shSTC1-5 (TRCN0000151758, TRCN0000154599, TRCN0000155141, TRCN0000156272 and TRCN0000157907 respectively), shCCR1-1 and 2 (TRCN0000008186 and TRCN0000008188 respectively) and shN.T (Catalog # SHC016). Lentiviral plasmid DNA was amplified from single bacterial colonies using Endofree Maxiprep kit Qiagen). For lentiviral infections, 1 million 293T packaging cells (ATCC) were transfected with 10 μg of lentiviral plasmid and 20μl (approximately 2μg) of Lentiviral Packaging Mix (Sigma Aldrich) in 10 cm dishes using the Profection® Mammalian Transfection System (Promega). After 48 hours, viral supernatant was collected, filtered, supplemented with 5 μg/mL polybrene and added to 1 million HFFF2 cells. This infection procedure was repeated after 6 hours but with 3 μg/mL polybrene (Millipore) and allowed to continue overnight. Selection was started 36 hours after the last infection, using puromycin (Sigma Aldrich) at 3μg/ml until the control (noninfected cells) died, approximately 72-96 hours. For direct transductions, 10,000 target cells (Cal51 or MDA-MB-231) were transduced with lentiviral particles at a multiplicity of infection (MOI) of 2.5 (calculated according to the manufacturer’s instructions). Knockdown efficiency was quantified using quantitative real time (qRT)-PCR analysis and/or immunoblotting.

**Quantitative RT-PCR and immunoblotting**

Quantitative RT-PCR was performed using the two-step procedure. In the first step, RNA from co-cultures, shRNA expressing HFFF2 cells or recombinant protein-treated fibroblasts was converted to cDNA by using the qScript® cDNA synthesis kit (Quanta Biosciences, CA). A total of 1 μg RNA was used for each reaction. In the second step, 0.5 μl of the resulting cDNA mix was used in a 20 μl quantitative PCR reaction using Perfecta SYBR Green Supermix with ROX® (Quanta Biosciences). Samples were amplified on an ABI7900 machine (Applied Biosciences, CA) using initial denaturation at 950C for 5 minutes followed by 40 cycles of amplification with annealing temperatures between 55 and 60^0^ C for 45 seconds. For each experiment, GAPDH expression was used as the normalization control and results were represented as percent expression relative to the respective control group. Reactions were performed in quadruplicate and analyzed using SDS 2.4 software (Applied Biosciences). Primers used are as follows (5’-3’):

AREG(Forward(F):TGGAAGCAGTAACATGCAAATGTC,Reverse(R):GGCTGCTAATGCAATTTTTGATAA),CCL2(F:CCCCAGTCACCTGCTGTTAT,R:TGGAATCCTGAACCCACTTC),CCL7(F:ATGAAAGCCTCTGCAGCACT,R:GGACAGTGGCTACTGGTGGT),CCL8(F:AGCCACTTTCAGCCCTCAG,R:CACAGCTTCCTTGGGACATT),STC1(F:TCAGCTGAAGTGGTTCGTTG,R:CAGCGCTGTACAAGAAGGATT), CCR1 (F: TTTGGTGTCATCACCAGCAT, R:GCCTGAAACAGCTTCCACTC), α-SMA (F: GTGTGTGACAATGGCTCTGG, R: TGGTGATGATGCCATGTTCT) and GAPDH (F: GAGTCAACGGATTTGGTCGT, R: TTGATTTTGGAGGGATCTCG). For immunoblotting analyses, cell lysates were prepared by scraping cells grown on 10 cm dishes using ice-cold RIPA buffer (Sigma Aldrich) containing Complete ® Protease Inhibitor Cocktail tablets (Roche, CA). The lysates were rotated at 40C for 30 minutes and supernatant collected after centrifugation at 14000 rpm for 30 minutes at 40C. Protein quantification was performed using Bradford assay (Biorad, CA) and 40 micrograms protein were used for each immunoblot lane. Immunoblotting was performed using standard procedures. Antibodies used were: Amphiregulin (ab89119; Abcam); CCL2 (ab9669; Abcam); CCL7 (ab18694; Abcam) and CCR1 (ab89527; Abcam) and β-actin (ab8224/8229; Abcam). Immunoblots were visualized using IR- 680 or IR-800 (LICOR Biosciences, NE) dye conjugated secondary antibodies. Membranes were scanned using Odyssey Scanner (LICOR Biosciences).

**Boyden Migration and scratch wound healing assay**

For Boyden migration, sub confluent wild type mouse embryonic fibroblasts (WT-MEFs) were incubated in DMEM+1% calf serum overnight. Subsequently, they were trypsinized, washed, counted and 100,000 cells added on the upper chamber of a 24-well plated fitted with a Boyden insert plate (Cytoselect, Cell Biolabs Inc., CA). DMEM supplemented with recombinant Amphiregulin (0, 50, 100, 200 ng/ml; Catalog # 262-AR-100, R&D systems, MN) was added to the lower chambers. The plates were incubated at 370C and cells were allowed to migrate for 5 hours. Post incubation, the upper chamber was transferred to an empty 24-well plate. The media was removed and the wells were washed in PBS following which the non-migratory were wiped off thoroughly with Q-tips several times. The migrated cells on the underside of the membrane were fixed with 100% ice-cold methanol for 10 minutes at room temperature and stained using 0.5% crystal violet solution (Sigma Aldrich) for 15 minutes at room temperature. Membranes were washed in distilled water and allowed to dry after which they were examined under high power objective (20X) on an Olympus FX70 microscope. Migratory cells in five different fields from each well were counted and averaged over four replicates. Each experiment was repeated three times. The results were presented as number of migratory cells/high power field. For the scratch wound healing assay, 100,000 wild type-MEFs were plated in 24-well plates in DMEM+ 1% calf serum (CS) and allowed to adhere overnight. Subsequently, a scratch wound was made using a clean, sterile plastic pipette tip in the center of each well. DMEM+1% CS supplemented with recombinant amphiregulin (100 ng/ml) was added to scratched WTMEFs. The plate was incubated at 370C during the course of the assay. Imaging of the scratch wound was performed using low magnification (4X and 10X) on an Olympus IX70 microscope, 0 hour and 16 hours post scratch. Images collected were imported into ImageJ software (NIH). Wound closure was calculated as the difference in the wound area between 0 and 16-hour time points and expressed as a percentage of control (0 ng/ml AREG). Experiments were performed in triplicate wells and repeated three times.

**BrdU proliferation, anoikis and MTT assays**

For comparisons of proliferative rates of HFFF2 and WT-MEFs treated with recombinant amphiregulin, 10000 cells were plated in 96 well plated and serum starved (DMEM+1% CS) for 48 hours. Recombinant amphiregulin (0, 10, 50, 100 and 200 ng/ml; R&D Systems) was added to the cells for 24 hours. Subsequently, proliferation rates were assessed using BrDU Cell Proliferation ELISA kit (Catalog # 11647229001; Roche) as per the manufacturer􀁠s instructions. Absorbance at 450nm was used as readout of BrDU incorporation. For comparisons of viability of cells expressing shRNA to the non-target (shRNA) (Cal51, MDA-MB-231 and HFFF2), or for breast cancer cells treated with recombinant AREG (R&D Systems), 10000 cells were plated in each well of a 96-well plate and cultured for 72 hours. MTT ((3-(4,5-Dimethylthiazol-2-yl)-2,5- diphenyltetrazolium bromide) assays were performed using MTT cell proliferation kit (Catalog # 11465007001; Roche) as per the manufacturer􀁠s instructions. Absorbance at 560nm was used as readout for viability. Results were expressed as percent absorbance compared to the respective control in each case. Anoikis assays were used to determine if treatment with recombinant amphiregulin (50, 100 or 200 ng/ml; R&D Systems) impacted the survival of Cal51 cells under anchorage independent conditions. Anoikis assays were performed by plating 10,000 serum starved (DMEM+1% CS) Cal51 or MDA-MB-231 cells in 96-well low-binding plates (Nalgene, CA) with recombinant amphiregulin (10, 50 or 100 ng/ml; R&D Systems). After 24 hours viability was determined by staining the cells with a fluorescent dye: 4 μM calcein AM (stains live cells; Life Technologies, CA) for 30 minutes at room temperature. Fluorescence intensities for live cells were determined using wavelength readings at 485nm on fluorescent plate reader (Victor 3, Perkin Elmer). Fluorescence intensities were represented as a percentage of the control (0 ng/ml AREG).

**Statistical tests for biological assays**

For determining if differences in tumor volume, immunostaining and cell-based assays between control and experimental groups were significant, a non-parametric, two-tailed Student’s t-test with a cutoff of p < 0.05 was used.

**Supplemental References**

Haibe-Kains, B., Desmedt, C., Loi, S., Culhane, A. C., Bontempi, G., Quackenbush, J., and Sotiriou, C. (2012). A three-gene model to robustly identify breast cancer molecular subtypes. Journal of the National Cancer Institute *104*, 311-325.
